# Managing ethical uncertainty: implicit normativity and the sociology of ethics

**DOI:** 10.1111/1467-9566.13010

**Published:** 2019-11-21

**Authors:** Alan Cribb

**Affiliations:** ^1^ Centre for Public Policy Research King's College London London UK

**Keywords:** ethics, uncertainty, health service organisations, professionalism

## Abstract

This article illustrates and discusses the idea of ‘implicit normativity’, and specifically its relevance to the management of ethical uncertainty. In particular I consider (i) the role implicit normativity plays in masking and containing potential ethical uncertainty and (ii) the contrast and boundary between implicit normativity and ‘overt ethics’ where ethical contestation – as well as particular processes and agents – are highlighted as salient. Using examples I show how the idea of implicit normativity can be applied not only to specific practices but also to whole fields of practice. The notion of ‘moral settlements’ – along with the explanatory role of the threat of ‘chaos’ – is introduced and elucidated to develop these points. I argue that attention to the management of ethical uncertainty shows the critically important contribution that an ambitious sociology of ethics can make to clinical ethics, including by helping to formulate and drive home questions about the ‘ethics of ethics’. The account presented here has resonances with work that seeks to use sociological lenses to move beyond conventional bioethics, including Petersen's (2013) call for a ‘normative sociology’.

## Introduction

The fact that health care operates in and creates normative spaces is very familiar, for example, from sociological analyses of biomedicine in general and countless specific critiques of healthcare policies and practices. What bioethicists have labelled as ‘implicit normativity’ – which also coincides with a central preoccupation of sociology – is a pervasive feature of health care. It has been elucidated by Carter (2018) as ‘the presence of unstated or taken‐for‐granted assumptions about what is good and bad, right or wrong, required or not required’. In principle any instance of implicit normativity could become an explicit concern within organisational or professional ethics and thus the focus of ongoing ethical contestation, but in practice this would hugely increase levels of uncertainty – cognitive and practical as well as ethical – in the system.

In this paper I will build on literature from bioethics and the sociology of ethics to discuss implicit normativity, concentrating on its role in the management of ethical uncertainty. In particular I will consider (i) the role implicit normativity plays in masking and containing potential ethical uncertainty and (ii) the contrast and boundary between implicit normativity and ‘overt ethics’ where ethical contestation – as well as particular processes and agents – are highlighted as salient. Ideas about implicit normativity, I want to suggest, can be applied not only to specific practices but also to whole fields of practice – to what I will call ‘moral settlements’. I argue that attention to the management of ethical uncertainty highlights the critically important contribution that an ambitious sociology of ethics can make to clinical ethics. Specifically, I suggest, such sociological work can support professional reflexivity by driving home questions about the social constitution of ethics including questions about the ‘ethics of ethics’. I argue that the more we can strengthen analyses of both the containment and the production of ethical contention the better placed we are to ask more penetrating questions about the way overt ethical debates and processes arise – what and who is made salient by them – including, for example, linked questions about accountability and blame. The account presented here has strong resonances with work that seeks to use sociological lenses to move beyond conventional bioethics (e.g. Frith 2012, Haimes 2002, Hedgecoe 2004, López 2004, Paton 2017) and especially Petersen's (2013) advocacy for a ‘normative sociology’ in the area of health.

The article is organised in four sections. First, I introduce and summarise the theoretical analysis – highlighting the crucial importance of managing ethical uncertainty in clinical systems and the role that moral settlements play in containing uncertainty. Second, I locate this analysis within important currents of related scholarship that help illuminate the critical contribution that sociology has made, and can make, to ethics. Third, I reflect upon examples to illustrate the practical relevance of the theoretical analysis to substantively important healthcare debates and to clinical professionalism. Fourth, I highlight the further scope for work that supports critical reflexivity in clinical ethics, including analyses of the ‘ethics of ethics’ as part of a call for further developments in normative sociology.

## Ethical uncertainty and moral settlements

Ethical uncertainty is potentially a very destabilising form of uncertainty in health care. This is both because it is a pervasive risk and because it is one that is very difficult to resolve. Ethical uncertainty cannot be eliminated by resolving (even imagining that we could) the factual dimension of uncertainty (summarised as a cluster of complications in the form of ‘probabilities, ambiguities or complexities’ by Han *et al*. 2011). Ethical theories, approaches and outlooks are inherently contested such that we could know everything else that we would like to know but still not know how, or have a basis for confidence about how, to proceed ethically. Almost anything could be turned into a set of live ethical issues and given that these are very unlikely to be explicitly answered to everyone's satisfaction such potential uncertainty needs to be managed. In this article, I am arguing that only some of this uncertainty is deliberately and self‐consciously managed through professional ethics, or other overt ethics discourses, but that much is implicitly managed through forms of social organisation and routine practice. In what follows I will be considering the relationship between the surface features of ethics and those that are ‘under the surface’ – between ethics as overt and as hidden – and in particular at how (what I am calling) ‘moral settlements’ manage ethical uncertainty partly by keeping it below the surface and partly through processes of naming, shaping and allocating those ethical agendas that do rise to the surface.

The pervasiveness of ethical uncertainty is very well captured in Greenhalgh's account of four facets of clinical uncertainty – (1) Evidence‐based medicine (EBM) type uncertainty about (probabilities) of benefits and harms in relation to diagnosis, referral, and treatment(s); (2) uncertainty surrounding a patient's biographical and illness narrative; (3) uncertainty in case‐based reasoning; and (4) uncertainty generated by the complexity of the broader inter‐professional and system context (including its technical manifestations and affordances) (Greenhalgh 2013). As Greenhalgh recognises, each of these facets – which co‐exist and interact – has an ethical dimension and facets 2 and 3 are explicitly discussed, in her account, in the language of ethics, as respectively about the engagement of the clinician with the patient's life and experiences and the business of helping to decide what it is best to do circumstance by circumstance. This emphasis is apposite in relation to the consciousness and agency of clinicians – it is largely where and how they can enact their ethical work. But ethical uncertainty is only partly managed through clinical ethics (at least if this is narrowly understood as about specific engagements with and decisions about/with patients) but is also, and first and foremost, managed through the construction of moral settlements.

In other words, to understand ethics – just as with health and illness – it is necessary to turn to social context. This insight is, for example, woven into Greenhalgh's account of clinical uncertainty – the experienced clinician will aim to find and navigate the best path in the circumstances but these circumstances will mean that some paths are more thinkable, visible, available, feasible and acceptable than others. And this fact is a function not just of the ‘values and preferences’ of individual patients or clinicians but of local and system‐wide norms, constraints, pressures and priorities. For example: How far are the recommendations of EBM incentivised or otherwise built into working conditions? Is the desire to practise a judicious integration of EBM and narrative medicine – a possibility highlighted by Greenhalgh – supported or hindered by prevailing routines? What values and priorities are made salient, or obscured, by the socio‐technical assemblages that partly constitute the clinical realm? To ask these questions is to begin to investigate the implicit normativity of, amongst other things, practices, cultures and technologies. In other words, ethics, here as elsewhere, is about deliberations and decisions but it is also about dispositions (including the ‘dispositions’ of non‐human actors, Gardner and Cribb 2016) and the overall ‘ethos’ they produce.

I am using the notion of moral settlement as a heuristic to refer to particular local versions of the social context of clinical ethics – just described with the shorthand ‘ethos’. Ethos here means something like the ‘character’ of a field of action (over and above the character of individuals), constituted by the totality of the socially embedded dispositions of human and non‐human actors. Just as with the more familiar term ‘policy settlement’ (or even geographical settlement) there is no implication that a moral settlement is planned, coherent or particularly stable, but it is simply the current constellation of influential norms and dispositions co‐existing in a complex amalgam. Clearly the term moral settlement picks out something relatively vague – a field that can only be characterised in contested ways and with fuzzy boundaries – but the point of deploying it here is not as a precursor to operationalisation but primarily to draw attention to the way that implicit normativity is not just a characteristic of specific practices, tools or measures but of social fields considered as a whole.

This can be seen by considering a seminal piece of research on implicit normativity by Molewijk *et al*. (2003). The focus of that work was a specific intervention but it also serves to open up the way normativity is built into the social field from which the intervention arises. Molewijk and colleagues focus on the development of an EBM decision‐support computer model and accompanying patient‐facing brochure (concerning the treatment of abdominal aneurysm). Faced with the question of how the ‘scientific facts’ can best be presented as a pre‐condition for supporting patient autonomy the researchers conclude that patient autonomy does not and cannot ‘come after’ or ‘follow on’ from the decision‐support tool but is already shaped by it. More generally they argue, ‘both the production and presentation of scientific information in evidence‐based decision‐support contain implicit presuppositions and values, which pre‐structure the moral environment of the clinical process of decision‐making. As a consequence, the evidence‐based decision support did not only support the clinical decision‐making process; it also transformed it in a morally significant way’. (p. 68)

This example helps me to develop this discussion of implicit normativity in three key respects. First, it is evident in this case that it is not merely the specific tool that is operative; rather the tool embodies and represents the logic and norms of EBM and it is these that ‘pre‐structure’ the moral environment (or at least it is these that are highlighted in this example – there are many other analogous factors; some indicated below). Second the ‘ethical work’ done by EBM (or analogous factors) – were it not for Molewijk and colleagues inviting us to look at and articulate it – is done under the surface or ‘in disguise’. That is, here it is done as part of a practical or technical agenda which is typically seen as outside and parallel to, or supportive of, ethical agendas. Third, what can be read as EBM tools obscuring ethical concerns could therefore, as well or instead, be read as such tools reducing or ‘containing’ ethical uncertainty.

When, in general, developers or users of EBM tools pay little or no attention to their more hidden ethical dimensions and effects – concentrating instead on more manifest ethical agendas to do with data‐related rigour, communicative clarity and the voluntariness of patient engagement – something important may be lost in relation to ethical sensitivity and rigour but arguably something is achieved in terms of the management of uncertainty. This suggests that there is, in broad terms, a ‘trade off’ for clinical professionals and other practical actors in the health system – the more that implicit normativity is made explicit the greater the danger that the lived experience of ethical uncertainty will become difficult to manage.

More broadly this example of an EBM tool is but one instance of many similar cases (familiar from the sociology of bioethics) where specific ethical issues are made salient whilst other ethical issues recede into the background, such that issues that are potentially contentious become invisible or are treated as natural or neutral. In this case, ‘shared decision‐making’ – which can be advocated as an ethical ideal – is supported whilst, at the same time, other ethically relevant considerations such as how easily patient autonomy can be colonised and circumscribed by biomedical reasoning are obscured under the guise of technical neutrality (Kelly *et al*. 2015). The idea of implicit normativity can thus reasonably be extended to those cases where there is already explicit reference to and consideration of ethical norms but where less overt norms are also ‘packaged in with’, or produced as side‐effects of, those overtly specified.

To talk about moral settlements is simply a summary way of highlighting the variety of respects in which, in Molewijk and colleagues’ terms, the moral environment is pre‐structured. As is probably evident there should be no equation between ethical norms being relatively settled and them being ‘right’ or ‘good’ (or, for that matter, ‘wrong’ or ‘bad’) – the issue in question is simply whether the norms in question are (more or less) the subject of contention or active debate. Similarly, it is important to stress that the management of ethical uncertainty should not be lazily valorised – that is, seen automatically as either a bad thing (obscuring important ethical questions) or a good thing (preventing an explosion of contention). If and when we are interested in pursuing substantive debates about what is right/good then we clearly need to consciously ‘stand back’ and question – reflexively and analytically – whatever is normally taken for granted and doing so is the staple both of philosophical bioethics and the ‘moral craft’ of professional or practical ethics (Boyd 2018, Parker 2015). The (additional) purposes of identifying and analysing moral settlements sociologically include the illumination of the large range and significance of relatively hidden factors that might otherwise be neglected, and the potential harnessing of the extensive critical and normative theoretical resources that sociology brings to this reflexive process.

This is not the place to enumerate the multiple forms and dimensions of environmental pre‐structuring produced by implicit normativity (although some indicative examples are offered in “Ethics in practice – both emergent and submerged” section of this paper). But a snapshot can be provided simply by noting down some of the more often recurring themes in analyses of healthcare norms. A clinician will be situated within a moral settlement that, for example, privileges certain constructions of healthcare purposes and methods (including certain constructions of biomedicine, as in EBM); embodies certain organisational forms and associated ideologically inflected values and virtues (such as more or less marketised, bureaucratic or ‘third sector’ forms and values); is to some degree ‘raced, classed and gendered’ (or, more broadly, fails to be responsive to the full spectrum of axes of social difference); is tacitly privileging some dimensions of quality over others (such as efficiency over person‐centredness); and is setting priorities and interpreting fairness on specific constructions of justice (e.g. focussing on the distribution of goods and outcomes, potentially at the expense of recognitional and participatory conceptions of justice (Fraser 1998)). And – as was illustrated in the discussion of Greenhalgh's work above – the relevant complex of norms that emerges through such factors is not simply to be understood as producing a background ‘climate’. Rather it will, to varying extents, be embedded in the field of practice – most crudely through things such as performance evaluation and guidelines but in many less crude ways also – and be constitutive of possibilities and tendencies in the enactment of clinical ethics.

## Understanding moral settlements – the sociological contribution

The idea of moral settlements is closely related to concerns explored by other scholars in both bioethics and the sociology of ethics. Leading figures in feminist bioethics have urged analysts to dig beneath mainstream narrow conceptions of healthcare ethics. For example, Jackie Leach Scully has helpfully summarised a core job of feminist ethics as about having regard to often neglected structural features of the ethical landscape – power structures, relationality and care, embodiment and marginal voices (2017). Scully also cites the pioneering work of feminist philosopher Margaret Urban Walker (2008) challenging the preoccupation of mainstream medical ethics with specific ethical decisions (and debates about their rationality or justification) when we should instead be focussing on the constitution of the ‘moral worlds’ that such decisions sit within – how is ethics produced, framed, sustained and adapted as a more or less collaborative set of social practices?

Striking parallels can be drawn between Walker's account and more recent work on the ‘new sociology of morality’ (Hitlin and Vaisey 2013). Hitlin and Vaisey argue that the object of sociology of ethics must be ‘thick morality’ not ‘thin morality’. Thick morality ‘focuses not only on single acts but also on moral identities and practices on a longer time scale and as they become a part of social institutions’ (p. 55). In their crystallisation of the distinctive contribution of sociology of ethics Hitlin and Vaisey specify that: ‘sociologists investigate the social processes that create and sustain particular conceptions of morality’; and ‘rather than primarily … investigating moral influences on judgements and single actions, sociologists seek to understand how morality affects strategies of action over time and/or in natural contexts’ (p. 54). In short, scholars from different disciplines have called for sociological study of the ways in which overt ethics ‘overlays’ and emerges from, underlying social and institutional fields and practices.

These two sets of authors also show how the production of moral settlements is inherently linked to the management of ethical uncertainty. Walker sees ‘moral worlds’ as the (often untidy and uncomfortable) products of sets of ongoing, evolving, social practices – they arise because we need ways of collectively living together, of protecting the things we care about, and in so doing making and coming to understand our self‐ and shared identities, including our expectations of one another. Through forging and defining our mutual responsibilities we become intelligible to ourselves and each other. Ethics ‘above the surface’ is roughly about deliberating and debating about specifics; ethics ‘under the surface’ provides a workable platform, a home, and shared frameworks from which to proceed. Of course – as Walker and other feminist philosophers are acutely aware – not everyone is equally placed to contribute to these shared frameworks, nor do they serve the interests of everyone equally well. Hence the importance of critiquing moral settlements. But, on the other hand, not every aspect of a framework that is problematic or contestable can be raised and challenged in every moment. The very possibility of practice depends on accepting some background set of local assumptions.

Again, Hitlin and Vaisey (2013) make some closely analogous points. They explain the substantial diminution in the study of morals within sociology (which they also illustrate quantitatively) – following a long period of it being central from the founding figures to Parsons – by reference to the widespread rejection of Parsons’ functionalism. Given Parsons’ influence an interest in morality came to be associated with conservatism, conformity and presumptions about the existence of universal, or at least societal‐wide, values. There is a substantial and legitimate worry that talk about the need for ‘shared frameworks’ can simply amount to re‐endorsement of the status quo and the accompanying reproduction of power hierarchies. But Hitlin and Vaisey are in fact arguing that a sociology of ethics can and should study ‘moralities’ – plural – both as things that exist and shape action and as things that are contested from inside and out and that are amenable to change: “Morality belongs more to cross‐cutting groups and less to society as a whole … Morality can bind groups together but it can also be the subject of negotiation, contestation, and exclusion” (p. 53, 54). These authors, in short, call for attention to the production and coalescence of the normative frameworks that lie beyond and underpin overt ethics.

As discussed above in relation to EBM decision‐support tools, social practices and arrangements can express and support explicit ethical concerns whilst, at the same time, ‘dampening down’ or obscuring other ethical concerns. Daniel Chambliss (1996) in his ground‐breaking account of the social organisation of nursing ethics provided some useful lenses for understanding these processes.

Much of Chambliss's analysis focuses on the ways in which the moral settlements of the hospitals he studied displace or disguise potential ethical issues – not only matters which would seem dramatic and obviously contentious outside hospital but matters which would be worthy of ethical scrutiny by nursing staff but which just ‘pass by’ or are ‘passed over’ (e.g. violations of privacy potentially going beyond what is strictly needed, or tests and procedures arguably, on occasions, causing more distress or pain than is warranted). In particular, he highlights sets of ‘routines’ and ‘rituals’ that shape ongoing practice, providing momentum and a sense of clarity about direction whilst thereby obscuring potential ethical issues: ‘The moral ambiguity of any one task becomes utterly lost in the pile of repeated events; the routine blurs the moral difficulties’ (p. 12). When Chambliss talks about ‘routines’ he is primarily talking about those organisational and professional regimes and practices that have coalesced into working norms, or ‘custom and practice’. But the idea that ‘routines’ can blur ethical issues lends itself to being extended to all of the human and non‐human dispositions that make up a moral settlement – whether these are embedded in habitual professional practices or in managerial systems, protocols and pathways, organisational measures, or tools and technologies.

For Chambliss ethics is ‘folded into routines whose aim is to maintain order’ (p. 180). He draws upon Goffman's work on ‘identity maintenance’ (in ways that corresponds with Margaret Urban Walker's work) – underlining the intrinsic connection between a degree of order and identity formation and continuity. Furthermore, he returns to the importance of identity maintenance when he moves on to consider the organisational production of ‘overt’ ethical issues: ‘Sometimes, though, the routine is broken; sometimes the moral taken‐for‐grantedness of the nurse's world is challenged. Problems arise, issues of right and wrong that cannot be covered by the usual assumptions. These problems strike at the heart of one's identity; they raise questions: “Who am I?” and “What sort of person will I be?”. Some such issues become conscious and visibly thought about, and nurses come to see them as ethical problems’ (p. 90). Chambliss offers a ‘structural theory’ to explain how issues pass through the boundary – which is normally maintained by routines – between tacit and explicit ethics. His analysis focuses mainly on the management of conflicts or tensions between organisational and professional interest groups such as nurses and doctors – for example, structural tensions between such things as attitudes to aggressive treatment, or about the relative importance of ‘treatment’ versus ‘care’ – these also being tensions which can be intensified with shifts in system and organisational power and status gaps over time. These examples indicate that ‘order’ in this context is neither abstract nor neutral but is likely to be entangled with historic hierarchies and vested interests.

Sociological work on the threat of chaos, therefore, does not only illuminate the management of ethical uncertainty but also the closely related questions raised by the ethics of uncertainty. Uncertainty is not only an epistemic concern but an existential one. This can be seen by starting from a site where the ethics of uncertainty has had considerable attention; that is, the consultation. How should a clinician who is navigating uncertainty – whether mainly about facts or values or a clear combination of both – handle this uncertainty as part of the consultation? Writing in 1997 Christopher Adamson, drawing on his own illness experience, analysed this issue in terms of the many potential interactions between, including ‘trade‐offs’ between, clinical uncertainty and existential uncertainty. In his account, ‘clinical uncertainty’ is an epistemic concept relating to the, more or less, incomplete knowledge of clinicians (including inevitable ‘shortfalls’ in biomedical sciences in general and in relation to specific cases) and ‘existential uncertainty’ is a phenomenological or affective concept relating to the experience of the patient. I would suggest that these two concepts can be detached from these two role positions and can both be seen as of relevance to all participants in healthcare systems. Navigating through the uncertainty of healthcare work and working environments is both about managing uncertainty for other people and about steering paths which enable actors to function effectively and to protect their own self‐identities. Given the potential impact of uncertainty on core aspects of identity and well‐being, the management of uncertainty can be seen as one of the most widespread and profound ethical challenges we face individually and collectively. This means, for example, that health professionals who are accountable not only for enacting health care, often in non‐ideal circumstances, but also for sustaining the broad effectiveness of the systems within which they operate, are not in a position to be continuously and pervasively ‘surfacing’ implicit normativity. It also means that they may need support when, in Chambliss's terms moral ‘taken‐for grantedness’ is challenged.

Of course we cannot invoke the threat of existential uncertainty, or the value of identity maintenance, as some kind of blanket rationale or justification for burying or hiding from uncertainty or for managing it thoughtlessly or defensively. The residual functionalism implicit in some of the *explanations* of moral settlements – for example, that the frameworks these represent (the assemblages of routines and dispositions etc.) are a pre‐condition for shared social practices – should not be taken to mean that any version of implicit normativity is acceptable as long as it wards off chaos. We can accept that these accounts have some broad explanatory merit but still insist that the substantive questions of ethics that relate to them remain completely open.

## Ethics in practice – both emergent and submerged

The argument of this article, in summary, is that ethical uncertainty is managed at two levels. There is the familiar face of ethics ‘above the surface’ – including the individual and collective work of professional ethics, codes of ethics, research ethics procedures (REPs), hospital ethics committees, ethics consultations and so on. But, working simultaneously, there are the ways in which ethical uncertainty is contained, and ethics is framed, by the implicit normativity of practices and broader settlements.

This can be illustrated by using the most familiar example of overt institutionalised ethics –REPs. REPs manage ethical uncertainty by containing the potential amount and diversity of clinical research activity (especially risky activity) and they filter and help structure and limit ethical uncertainties within specific projects. As well as providing important safeguards (and, of course, legal protection) for institutions they provide shared frameworks and ‘ways of going on’ for researchers and participants. REPs allocate accountabilities to certain agents and determine and delimit the ‘rules of the game’. They comprise carefully developed and specified architectures and tools for managing specific ethical issues (most conspicuously consent procedures), are populated with specific roles – gatekeepers, reviewers, advisers, chairs – and produce definitively worded socially authorised decisions (O'Reilly *et al*. 2009). However, most researchers have a clear and strong sense that institutionally defined ‘research ethics’ is something much narrower than the ‘ethics of research’. In other words, they recognise the danger that, in practice, many important ethical questions lie beyond, and are relatively sidelined by, the emphases of REPs. These include, for example, important questions about the influence of hierarchy, power and trust whether at a micro‐level, such as in actual enactments of consent (Armstrong *et al*. 2012, Brown *et al*. 2015, Corrigan 2003) or at a macro‐level, such as in relation to the possibilities and challenges of genuinely emancipatory forms of patient and public participation and co‐production in research (as analysed by Prainsack 2017).

This situation is mirrored in clinical ethics. As outlined in the “Ethical uncertainty and moral settlements” section, uncertainty in clinical ethics is partly managed by individuals consciously navigating through the judgements of professional ethics but this conscious process takes place in a context which shapes and limits what is possible and what is made salient (here it is tempting, of course, to shamelessly re‐work and re‐purpose Marx and say ‘clinicians enact their own ethics but they do not enact them as they please; they do not enact them under self‐selected circumstances, but under circumstances existing already’). There are few close analogues to the strong architecture of research ethics (such as resource allocation committees) but there are countless ways in which moral settlements frame and ‘perform’ ethics including through institutional norms and priorities, whether these are explicitly articulated or embedded in structures, policies, practices, tools, measures and so on. The implicit normativity of moral settlements operates at both large and small scale, and can be seen in relation both to overt ethics practices and less overt cases. Here I can only offer a few token examples of a pervasive phenomenon.

The policy effects of economic context provide a familiar and clear example of a large‐scale contribution to moral settlements in which ethical relevance and influence is often hidden. For example, Kerasidou (2019), has recently used fieldwork in English Accident and Emergency hospital departments to uncover the detrimental impact of economic austerity on professional empathy and compassion, arguing that policy technologies ‘are not only responsible for redesigning the structure and form of healthcare systems but also carry profound normative implications, which should not be ignored or overlooked. By using incentives and punishment, governments and healthcare systems control the way healthcare professionals perceive and enact their duties and obligations in practice. This way, these policies create the environment in which what counts as “right action” is (re)defined’. Part of this kind of scholarly work is thus concerned with uncovering the ways in which what appear to be practical contingencies contribute to ethics. The full ethical implications of insufficiently examined efficiency measures were notoriously unpacked in the Francis Inquiry Report on the failures at Mid Staffordshire NHS Foundation Trust, where an uncritical and inflexible leadership approach to target‐driven priorities emerged as a major contributor to serious care scandals (Francis 2013).

Although the issues raised in the Francis Inquiry Report were extreme they are illustrative of how ethical issues can be contained and submerged but then emerge vividly into the light, sometimes as scandals. As noted above we can extend Chambliss’ notion of ‘routines’ to apply to any organised pattern of social activity, pathway, tool‐kit or similar. Routines help manage uncertainty but thereby risk diverting or mis‐directing ethical attention. In recent years there have been a number of very high‐profile cases in the UK where widely accepted health service ‘custom and practice’ has become a site of heightened and substantial ethical contention and controversy. The debates surrounding the phasing out and replacement of the palliative care approach known as ‘the Liverpool Care Pathway’ (LCP) will serve as an example (see National Health Service 2012, 2015). Such high‐profile cases simply represent one set of, albeit especially dramatic and public, examples of how the ethical dimension and contestability of healthcare practices can move from being relatively hidden to being visible (and often back again). Ethical uncertainty can be circumscribed and managed, can then surface and even become ‘out of control’, and can thus become a locus of explicit ethical debate such that official procedures are harnessed or put in place to manage the overt ethical concerns and dilemmas that have surfaced. In the case of LCP the potential uncertainties related to what had become ‘routine’ procedures in end‐of‐life care, which were managed through the LCP practice guidelines that had been trialled, developed and eventually endorsed nationally but which later became an arena of debate (first as a source of professional and academic contention and then through broadcast and social media advocacy and controversy); this then produced formal processes of review and new guidance to ‘re‐contain’ the uncertainties. (For those who are unfamiliar with LCP related debates, Seymour and Clark 2018 offer an excellent account of the ‘rise, demise and legacy’ of the LCP, along with the historical and ongoing axes of controversy around it).

The many kinds of instances I have in mind are much more commonplace and (typically) much less contentious and public than these high‐profile kinds of cases but they embody an analogous logic. It is worth noting, for example, that Stacy Carter, with whose definition of implicit normativity I began, presented this definition within a discussion of healthcare quality improvement in which she showed how all components of quality improvement rely on implicit, taken‐for‐granted norms which could (and often should) be surfaced and subjected to ethical analysis (Carter 2018). The decision‐support tool (Molewijk *et al*. 2003) discussed in the first section of this paper, as part of the introduction of implicit normativity, is also a good example. This kind of tool is very unlikely to create a scandal. But such tools can serve to construct roles and relationships and, in a variety of other ways ‘pre‐structure’ ethics. Clinicians making use of them have their own goals and responsibilities partly configured by the processes and values embodied in the tools. Kocman *et al*. (2019), for example, show how the design of a quality improvement checklist can reflect different modes of clinical responsibility with contrasting, and sometimes conflicting, logics. Tools and measures, in other words, arguably embody sets of ethical dispositions and thus operate as ethical actors in their own right (for an extended example of this see Gardner and Cribb 2016).

There are also cases where the ethics functions of a tool are completely overt but where key dimensions and implications of the tool in use come to be reappraised and reworked, again without any major controversy, through a process of ethical reflection and innovation. An example is Fritz's work on ‘do not attempt cardiopulmonary resuscitation’ (DNACPR) forms. DNACPR forms in a hospital record specifically indicate that a DNACPR decision has been made either because the patient expressly does not want CPR or because it has little chance of success and a high chance of prolonging or causing suffering. However, research on the uses and interpretation of such forms shows ‘they have taken on practical, legal, and emotional significance far beyond their intended remit’. (Fritz *et al*. 2017) This includes evidence that clinicians may over‐interpret the presence of completed DNACPR forms and be less likely to give other kinds of treatment or palliative care, with the result that a patient's overall care can be seriously compromised. These kinds of findings encouraged Fritz and colleagues to develop new broader ranging forms, and accompanying processes, that better prompt and facilitate doctor‐patient communication and encourage consideration of a wider range of treatment possibilities within the context of attention to overall care goals. It is notable that this development is being carefully refined and reviewed precisely because its advocates have come to understand that such forms have an ‘ethical life’ of their own.

The moral settlements within which clinical work takes place are constituted by multiple overlapping forms of implicit normativity. The examples summarised here underline how the conscious efforts of clinicians to manage ethical uncertainty represent only a fraction of the processes that contain such uncertainty. In any case, as is well known, conscious efforts may be insufficient. For example, a clinician's motivation might, in a particular instance, be underpinned by concerns for community well‐being and solidarity but those concerns can be cancelled out by the much narrower individualistic and biomedical tools and measures that institutionally define their role. Clinical ethics is accomplished by clinicians and moral settlements in combination. These examples also illustrate the link between implicit normativity and the management of uncertainty – the more that the many values bound into moral settlements are made explicit the more that various forms of uncertainty will rise to the surface – especially as seen from the vantage point of practical actors, who need to work with complexity and do not have the luxury of merely contemplating it.

## Sociology and the ‘ethics of ethics’

A focus on ethical uncertainty in clinical contexts highlights the considerable potential for further work in, and developments in, sociology of ethics. Such developments may take a number of different forms – they could, for example, contribute to practical agendas at a professional or system level, to strategic shifts in the framing of ethical debates, and to more theoretical work including normative theorising. I will say a little more about each in turn.

As noted earlier, sociologists are well placed to illuminate the complex roles played by the countless number of factors that shape the diverse ethical landscapes of health care. The examples rehearsed in the previous section provide a small indication of the scope for already ongoing conversations between sociologists and clinicians (and others) to support ethical reflexivity in health care. A key activity here is, of course, to help clinicians and other health system actors ‘deconstruct’ the familiar category of ‘ethics’ in so far as this is treated as categorically distinct from other kinds of considerations – economic, technical, biomedical and so on. At the same time, as I have tried to underline, everyone with an interest in clinical ethics needs to be mindful of the additional, very significant, ‘uncertainty burden’ that can be generated by highlighting the role of implicit normativity.

In addition to the detailed study of how moral settlements and overt systems of professional ethics are socially produced sociologists can also help address ethical questions about these settlements and systems – what makes them better and worse, or more or less defensible – that is, can contribute to what might be called the ‘ethics of ethics’. There are, for example, fundamental questions about which ethical issues are surfaced and which relatively buried, who gets positioned as ethically significant agents and what forms ethical discourse take. As I have stressed not everyone is equally well placed to shape either the moral settlements or the overt ethical practices of health care – indeed these will tend to reflect, and thereby sustain, prevailing hierarchies and vested interests. Although both implicit normativity and overt ethics are socially produced some people have many more opportunities to affect, and hence much more responsibility for, the forms they take. Specifically, in relation to the management of uncertainty, for example, there are substantively important questions to ask: ‘Accepting that not all kinds of ethical uncertainties can be psychologically or socially managed at once, how open is the prevailing settlement to being critically reflexive about implicit normativity and what processes exist to enable, and to include a range of relevant actors in, such reflexivity?’. ‘How are those people who are living with and managing ethical uncertainty and who have to make difficult judgements in the context of uncertainty supported to do so?’.

These questions sound abstract but can be translated into practical questions relatively easily. One clear example is the advocacy by Vriens *et al*. (2016) of what they describe as a ‘conditional approach’ to professional accountability. Their argument largely inverts the currently dominant approach to accountability in public sector healthcare systems. Instead of concentrating on the mechanisms that have been put in place to hold clinical and other professionals accountable to the institutions that employ them – accountability mechanisms that often have both coercive and reductionist effects – Vriens *et al*. ask how institutions can be held accountable for providing the conditions needed for professionalism. That is, given that professional judgement necessarily involves a measure of creative autonomy in steering routes through many uncertainties in context‐sensitive ways, we can examine whether structural and cultural conditions, and practical support systems, are being provided to enable professionals to meet these challenges. Institutions should be able to ask themselves, and could be judged on the basis of, how far they are enabling and encouraging more critically reflexive forms of professional ethics rather than unthinking but ‘safe’ compliance. It seems reasonable to argue, for example, that audit mechanisms should not be in place to regulate professional ethics unless something equivalent is in place to judge the policymakers, system leaders and managers who create the possibilities of action. As is familiar from other areas of sociology of health we must be sceptical when blame is allocated to people operating in challenging conditions and not to those who help to create those conditions.

On the basis of this kind of example I would want to argue that sociological analysis and theorising in this area can inform normative and not just descriptive or explanatory thinking. One thing is clear – whilst sociological analyses can certainly help to explain the containment, production and shape of ethical concerns, practices and debates in clinical settings as well as elsewhere (e.g. Brosnan *et al*. 2013, Wainwright *et al*. 2006) this does not dissolve them away. Ethical agendas and dilemmas – although some of them may look different or take different forms post‐analysis – still need to be addressed. We can come to see how ‘invisible’ routines may carry undeclared ethical loads but we still need to decide whether and how to continue with, abandon or reform them. We may uncover and map the ways in which carefully articulated and designed tools, measures, protocols or care pathways often do both overt and more hidden ethical work. Such analyses provide us with a much richer account of what is at stake in the development and/or deployment of such tools etc. – including the many normative considerations that can be masked in surface accounts – but, once again, this simply makes the substantive ethical issues more challenging. We need to find ways of looking and working ‘in depth’ – working on the value‐judgements and dilemmas that are foregrounded and, simultaneously, on those hidden by the pre‐structuring of the moral environment. In other words, we need to do both sociology and ethics at the same time.

There are strong parallels between the line of argument I have developed with regard to clinical ethics and debates within sociology of bioethics. As noted in “Understanding moral settlements – the sociological contribution” section above – work in the sociology of bioethics has highlighted ethical issues that might otherwise be neglected and raised sceptical concerns about some of the dominant foci and approaches of bioethics. López (2004), for example, problematises not only the foci but also the social authority of conventional bioethics – suggesting that looking at the ‘cognitive content’ of bioethics does not help us understand why it is socially influential. Rather, he argues, dominant forms of bioethics borrow their authority by discursively aligning themselves with powerful discursive formations – in brief the law, cost‐benefit analysis, biomedicine and political liberalism. These discursive formations – it might be said, to echo, in summary, the argument of this paper – manage uncertainty and do so by circumscribing what is typically ‘sayable’. López's account also indicates that although I have sometimes referred to specific local moral settlements these are, in significant measure, constituted by more ‘global’ elements, that is, there are very influential features of moral settlements that stretch across many boundaries.

What is needed – as López and others (discussed in “Understanding moral settlements – the sociological contribution” section) have highlighted – is a much more expansive conception of ethics that takes into account and can tackle the ways ethics is produced. I would argue that this programme points in a direction that Petersen (2013) describes as ‘a normative sociology of ethics’ (or what some might like to describe as a sociologically critical empirical ethics, Ives *et al*. 2017). Petersen, as with other critics, challenges the relatively narrow gaze of much bioethics and calls for more attention to underlying structural questions, especially the power of global capitalism and its role in shaping bio‐knowledge and thus health opportunities and experiences beyond (as well as within) health care. I would want to strongly echo his call for a sociology of ethics to be normative as well as explanatory – and normative in two senses, both as having action‐guiding potential and as deploying normative (ethical and critical) lenses. Although, whilst emphasising this potential normative contribution, it is worth underlining the well‐rehearsed gap between sociological dispositions and the direct activity of normative prescription or guidance as, for example, represented and articulated so eloquently by Charles Bosk (2008).

Petersen develops and champions his own sociologically reworked human rights approach as a way of moving beyond bioethics. In making his case for a human rights approach he maps out the rich tool‐kit of other normative concepts and theories available to sociology from a range of critical disciplines (as well as usefully reviewing the range of philosophical lenses conventionally applied in ethics). I would see his account as a good platform for building a normative sociology of ethics, although I would want to stress that I think there is a place in a suitably ambitious sociology of ethics for drawing upon all of the theoretical tools he reviews. There is vast potential for interdisciplinary work that sits between sociology of health and ethics; and this includes foundationally important sociological contributions to what I am highlighting here as the ‘ethics of ethics’. If we are to understand the social production of ethical issues – including what is involved in ‘containing’ as well as overtly enacting ethics – and also contribute towards ethical resolutions at the multiple levels and for the very many diverse agents implicated, then we need all the help we can get from the full spectrum of philosophical and sociological resources.
